# Adipose tissue-derived stromal/stem cells + cholecalciferol: a pilot study in recent-onset type 1 diabetes patients

**DOI:** 10.20945/2359-3997000000368

**Published:** 2021-04-29

**Authors:** Joana Rodrigues Dantas, Débora Batista Araújo, Karina Ribeiro Silva, Débora Lopes Souto, Maria de Fátima Carvalho Pereira, Ronir Raggio Luiz, Matheus dos Santos Mantuano, Cesar Claudio-da-Silva, Monica Andrade Lima Gabbay, Sérgio Atala Dib, Carlos Eduardo Barra Couri, Angelo Maiolino, Carmen Lúcia Kuniyoshi Rebelatto, Débora Regina Daga, Alexandra Cristina Senegaglia, Paulo Roberto Slud Brofman, Leandra S. Baptista, José Egídio Paulo de Oliveira, Lenita Zajdenverg, Melanie Rodacki

**Affiliations:** 1 Universidade Federal do Rio de Janeiro Departamento de Nutrologia e Diabetes Rio de Janeiro RJ Brasil Departamento de Nutrologia e Diabetes, Universidade Federal do Rio de Janeiro, RJ, Brasil; 2 Universidade Federal do Rio de Janeiro Instituto Nacional de Metrologia, Qualidade e Tecnologia Laboratório de Bioengenharia de Tecidos Rio de Janeiro RJ Brasil Laboratório de Bioengenharia de Tecidos, Instituto Nacional de Metrologia, Qualidade e Tecnologia (Inmetro), Universidade Federal do Rio de Janeiro, Rio de Janeiro, RJ, Brasil; 3 Universidade Federal do Rio de Janeiro Serviço de Patologia Clínica Rio de Janeiro RJ Brasil Serviço de Patologia Clínica, Universidade Federal do Rio de Janeiro, Rio de Janeiro, RJ, Brasil; 4 Universidade Federal do Rio de Janeiro Instituto de Estudos de Saúde Pública Rio de Janeiro RJ Brasil Instituto de Estudos de Saúde Pública, Universidade Federal do Rio de Janeiro, Rio de Janeiro, RJ, Brasil; 5 Universidade Federal do Rio de Janeiro Faculdade de Medicina Rio de Janeiro RJ Brasil Faculdade de Medicina, Universidade Federal do Rio de Janeiro, Rio de Janeiro, RJ, Brasil; 6 Universidade Federal do Rio de Janeiro Departamento de Cirurgia Plástica Rio de Janeiro RJ Brasil Departamento de Cirurgia Plástica, Universidade Federal do Rio de Janeiro, Rio de Janeiro, RJ, Brasil; 7 Universidade Federal de São Paulo São Paulo SP Brasil Universidade Federal de São Paulo, São Paulo, SP, Brasil; 8 Universidade de São Paulo São Paulo SP Brasil Universidade de São Paulo, São Paulo, SP, Brasil; 9 Universidade Federal do Rio de Janeiro Departamento de Hematologia Rio de Janeiro RJ Brasil Departamento de Hematologia, Universidade Federal do Rio de Janeiro, Rio de Janeiro, RJ, Brasil; 10 Pontifícia Universidade Católica do Paraná Curitiba PR Brasil Core Cell Technology, Pontifícia Universidade Católica do Paraná, Curitiba, PR, Brasil; 11 Universidade Federal do Rio de Janeiro Rio de Janeiro RJ Brasil Centro Multidisciplinar de Pesquisas Biológicas (Numpex-Bio), Universidade Federal do Rio de Janeiro, Rio de Janeiro, RJ, Brasil; Laboratório de Bioengenharia de Tecidos, Instituto Nacional de Metrologia, Qualidade e Tecnologia (Inmetro), Rio de Janeiro, RJ, Brasil

**Keywords:** Type 1 diabetes, pancreatic function, adipose tissue-derived stromal/stem cells

## Abstract

**Objective:**

Adipose tissue-derived stromal/stem cells (ASCs) and vitamin D have immunomodulatory actions that could be useful for type 1 diabetes (T1D). We aimed in this study to investigate the safety and efficacy of ASCs + daily cholecalciferol (VIT D) for 6 months in patients with recent-onset T1D.

**Materials and methods:**

In this prospective, dual-center, open trial, patients with recent onset T1D received one dose of allogenic ASC (1 x 10^6^ cells/kg) and cholecalciferol 2,000 UI/day for 6 months (group 1). They were compared to patients who received chol-ecalciferol (group 2) and standard treatment (group 3). Adverse events were recorded; C-peptide (CP), insulin dose and HbA1c were measured at baseline (T0), after 3 (T3) and 6 months (T6).

**Results:**

In group 1 (n = 7), adverse events included transient headache (all), mild local reactions (all), tachycardia (n = 4), abdominal cramps (n = 1), thrombophlebitis (n = 4), scotomas (n = 2), and central retinal vein occlusion at T3 (n = 1, resolution at T6). Group 1 had an increase in basal CP (p = 0.018; mean: 40.41+/-40.79 %), without changes in stimulated CP after mixed meal (p = 0.62), from T0 to T6. Basal CP remained stable in groups 2 and 3 (p = 0.58 and p = 0.116, respectively). Group 1 had small insulin requirements (0.31+/- 0.26 UI/kg) without changes at T6 (p = 0.44) and HbA1c decline (p = 0.01). At T6, all patients (100%; n = 7) in group 1 were in honeymoon vs 75% (n = 3/4) and 50% (n = 3/6) in groups 2 and 3, p = 0.01.

**Conclusions:**

Allogenic ASC + VIT D without immunosuppression was safe and might have a role in the preservation of β-cells in patients with recent-onset T1D. ClinicalTrials.gov: NCT03920397.

## INTRODUCTION

Type 1 diabetes (T1D) is a chronic disease characterized by immune-mediated destruction of pancreatic β-cells requiring life-long insulin treatment. Preservation or recovery of residual β-cells could cure the disease (1-4) and avoid insulin requirements. Even a partial response, unable to induce cure, appears to be beneficial, leading to lower insulin doses and reduced frequency of both severe hypoglycemia and chronic complications (5).

Immunomodulatory and immunosuppressive agents have been tested to preserve β-cell function (6-8). Non-myeloablative transplantation of autologous hematopoietic stem cells (HSC) showed favorable results and exogenous insulin withdrawal (at least for a short period) in individuals with T1D, but it requires immunosuppression (9). Mesenchymal stem cells (MSCs) are potential alternatives due to their immunomodulatory properties without the need for immunosuppression (10,11). MSCs decrease proliferation and activation of natural killers (NK), dendritic and T cells, reduce secretion of inflammatory cytokines and may have antiapoptotic properties (12). Therefore, they may have a protective role in the autoimmune destruction of β-cells. Adipose tissue represents an abundant and easily accessible source of MSCs (13), which may be of great clinical interest.

Vitamin D is another potential immunomodulatory agent. In vitro and in vivo studies suggest that 25(OH) vitamin D inhibits lymphocyte proliferation and modifies the Th1/Th2 cytokine profile, which may reduce damage associated with the Th1 immune response (14,15). Gabbay and cols. have shown preservation of C-peptide (CP) secretion with 2,000 UI/day of vitamin D (VIT D) (16) in patients with recent-onset T1D. However, the benefits of VIT D supplementation are still controversial (17-19).

T1D has a complex pathophysiology that involves multiple immune pathways. Thus, it is probable that the ideal intervention for its cure would include a combination of drugs with different mechanisms of actions. The aim of this study was to investigate the safety and efficacy of adipose tissue-derived stromal/stem cells (ASCs) infusion + daily cholecalciferol (VIT D) for 6 months in patients with recent-onset T1D. We also performed a pilot analysis comparing these results with those obtained from a previous case control study that investigated the effect of solely VIT D supplementation in patients with T1D (16).

## RESEARCH DESIGN AND METHODS

### Patient selection and study design

This was a prospective, dual-center, open trial in which patients with recent onset T1D received one dose of allogenic ASC and VIT D 2,000 UI/day for 6 months. The sample was selected by convenience. Participants signed an informed consent. The study was approved by the Institutional Review Board (17488313.1.0000.5257, University Hospital Clementino Fraga Filho [HUCFF]) and registered at ClinicalTrial.gov (NCT03920397). Inclusion criteria were diagnosis of T1D according to American Diabetes Association (ADA) criteria for < 4 months; ages between 16 and 35 years, and positive glutamic acid decarboxylase antibody (GADA). Malignancy, infections, pregnancy, breastfeeding, renal dysfunction and diabetic ketoacidosis were exclusion criteria.

### Lipoaspirate human samples and ASC culture

Adipose tissue samples were obtained through liposuction of three healthy females. Donor's serology testing was negative for syphilis, Chagas disease, Hepatitis B and C, HIV and HTLV. Donors had Cytomegalovirus IgG+ with negative polymerase chain reaction (PCR) in blood samples and ASCs.

ASCs were isolated, cultured and characterized as previously described (13). Samples were processed at the Core Cell Technology facility of Pontifícia Universidade Católica do Paraná. Briefly, 100 mL of adipose tissue was washed in sterile phosphate-buffered saline (PBS) (Gibco Invitrogen). A one-step digestion by 1 mg/mL collagenase type I (Invitrogen) was performed for 30 minutes at 37 °C during permanent shaking, followed by a filtration step through a 100 μm mesh filter (BD FALCON, BD Biosciences Discovery Labware). The cell suspension was centrifuged at 800 g for 10 minutes, and erythrocytes were removed through a lysis buffer with pH 7.3. The remaining cells were washed at 400 g for 10 minutes and then cultured at a density of 1 × 10^5^ cells/cm^2^ in T75 culture flasks and DMEM-F12 (Gibco Invitrogen) supplemented with 10% of fetal calf serum, penicillin (100 units/mL), and streptomycin (100 μg/mL). The culture medium was replaced three days after seeding, and then twice a week. ASCs were subcultured after reaching 80% confluence, with 0.5% trypsin/EDTA (Invitrogen) solution. Cells were related at a density of 4x10^3^ cells/cm^2^ for expansion (13).

Quality control of cell suspension sterility was evaluated by tests to detect bacteria and fungi (Bact/Alert 3D, Biomerieux), endotoxins (Endosafe™ PTS, Charles River) and Mycoplasma (KIT MycoAlert™ PLUS Mycoplasma Detection, Lonza). Cell viability was performed by flow cytometry using the vital dye 7-AAD (7-Aminoactinomycin D – BD#559925) to determine the percentage of viable cells and Annexin V protein (BD#51-65875X) to determine the percentage of cells in apoptosis. Cytogenetic analysis was performed using the GTG-banding method.

Cells were phenotypically characterized by flow cytometry before the clinical application, using the following monoclonal antibodies: FITC-labeled CD14 (BD#555397), CD45 (BD#555482), CD19 (BD#555412), CD44 (BD#555478); PE-labeled CD73 (BD#550257), CD90 (BD#555596), CD166 (BD#559263), PerCP-labeled HLA-DR (BD#551375); APC-labeled CD34 (BD#555824), CD105 (BD#562408), CD29 (BD#559883) all purchased from BD (Pharmingen). At least 100,000 events were acquired on a BD FACSCalibur™ flow cytometer (BD Biosciences), and data were analyzed using FlowJo 10 (TreeStar) software (13) (Supplementary Material – Supplementary Table S1).

### ASC infusion

On the day of infusion, the ASC monolayer were dissociated as described above, and 1 x 10^6^ cells/kg of the recipient patient were resuspended in 5 mL of saline solution with 50% albumin and 5% ACD (Anticoagulant Citrate Dextrose Solution). The Cell suspension was sent to the hospital in a cooler with recycled ice.

Patients that received ASCs were admitted to the hospital on the day of the infusion and discharged 24 hours after infusion. A single dose of ASCs was infused in a peripheral upper arm vein for 15-20 minutes. Patients started taking oral cholecalciferol 2,000 UI one day after the infusion of ASCs.

### Safety tests

Adverse events were recorded during hospitalization and at each follow-up outpatient visit (T1, T3, and T6), with clinical and laboratory exams (blood count, lipids, renal and hepatic function, TSH, free tyroxine, anti-TPO, calcium, phosphorus and 25(OH) vitamin D, performed with automated biochemical equipment CMD 800 IX1).

### Clinical and pancreatic function evaluation

Participants were followed for 6 months. In the first visit (T0), all patients were interviewed and had a physical exam. Weight, height, body mass index (BMI), blood pressure, heart frequency, frequency of hypoglycemia and insulin dose/kg of body weight were evaluated at T0 and after 1 (T1), 3 (T3) and 6 (T6) months. Insulin dose adjustments were performed at each visit as necessary. Patients received nutritional guidance according to ADA recommendations (20). Blood samples were drawn at T0, T1, T3 and T6 for the following measurements: HbA1c (High Performance Liquid Chromatography by boronate affinity), blood count and biochemistry analysis, 25(OH) vitamin D (automated CMD 800 IX1), GADA (ELISA assay, Euroimmun brand and Molecular Devices Spectra max reader) and CP (Microparticle Chemiluminescent Immunoassay, Architect Abbott) before and 30, 60, 90 and 120 minutes after liquid mixed meal (Glucerna^®^). The area under the curve (AUC) for CP was calculated. Adverse events were recorded during hospitalization and at each follow-up visit.

### Comparison with previous case-control study using only VIT D supplementation as intervention

We compared our results with patients previously included in a case-control study that investigated the effects of a daily dose of 2,000 UI VIT D without ASC in individuals with recent onset T1D and similar age (>15 y/o), from a different population (São Paulo) in the same region of the country, Southeastern Brazil (16). Therefore, we established three patient groups for comparison: 1) ASCs + VIT D supplementation; 2) VIT D supplementation; 3) Conventional treatment. Group 3 comprised individuals from both centers: Rio de Janeiro (n = 2) and São Paulo (n = 4). Insulin therapy was prescribed for all patients. Dose adjustments were performed according to glycemic control. Changes in HbA1c, CP and insulin dose/kg were compared between groups. CP was analyzed by immunofluorometric assay (AutoDelfia) at T0 and T6, considering basal and peak stimulated CP after a mixed-meal test (MMT). AUC was not available for comparison.

### Statistical analysis

Data are expressed as mean ± standard deviation. Descriptive statistics have been used to summarize patients' characteristics. Comparisons of categorical variables were performed with Chi square test. A Wilcoxon test was used to compare results at baseline and after follow-up in each group. Continuous variables were compared using Kruskal-Wallis for multiple-group comparison and Mann-Whitney for two-by-two comparisons. A Spearman test was used to investigate correlation between continuous variables. Statistical tests are based on a 2-sided significance level of 0.05. For multiple comparisons, Bonferroni correction was applied and the significance level of 0.017 was considered. SPSS software, version 21.0 was used for statistical analyses.

## RESULTS

### Clinical characteristics of the study group

Eleven patients were interviewed, and two were excluded (one used glucocorticoid, and another had renal dysfunction). Nine patients were evaluated: seven received ASCs + VIT D and two were included as controls. All completed 6 months of follow-ups.

The mean age of patients that underwent intervention was 27.14 ± 6.49 years old; 3 were males, and 2 were non-whites. The T1D duration at T0 was 2.6 ± 1.03 months. Mean initial serum 25(OH) vitamin D was 33.06 ± 13.55 ng/mL, patients were not taking any vitamin supplementation prior to the study. The control patients were 16 and 20 years old. Clinical characteristics of the study group are described in Table 1.

**Table 1:** Clinical Characteristics of each patient in the ASC + VIT D group

	Age	Gender	Ethnicity	Body mass index	Disease duration	ASC concentration
Patient 1	26	Male	White	26.06 kg/m^2^	4 months	78 x 10^6^ cells
Patient 2	35	Male	Non-white	25.91 kg/m^2^	4 months	74 x 10^6^ cells
Patient 3	28	Male	White	23.38 kg/m^2^	2 months	65 x 10^6^ cells
Patient 4	34	Female	White	23.56 kg/m^2^	2 months	73 x 10^6^ cells
Patient 5	16	Female	White	20.96 kg/m^2^	3.5 months	55 x 10^6^ cells
Patient 6	23	Female	Non-white	20.76 kg/m^2^	1.7 months	60 x 10^6^ cells
Patient 7	28	Female	White	23.71 kg/m^2^	2 months	69 x 10^6^ cells
Patient 8	16	Male	White	18.25 kg/m^2^	2 months	Control
Patient 9	20	Female	Non-white	23,71 kg/m^2^	4 months	Control

kg: kilograms; m: meters; ASC: adipose tissue-derived stem/stromal cells.

### ASC infusion and adverse events

For ASC infusion, the mean number of cells was 67.71 x 10^6^, with 95.10% cell viability. Tests for a microorganism's growth control were negative. ASCs were immunophenotypically characterized as follows: CD105: 94.18%; CD73: 96.46%; CD90: 99.80%; CD29: 99.15%; CD166: 94.04%; CD44: 89.13%; CD14:1.94%; CD34: 0.59%; CD45: 0.87%; CD19: 0.71%; HLA-DR: 0.64%. No clonal chromosomal rearrangements were detected. Samples were approved by cytogenetic quality control for therapeutic use.

All patients had transient headache and mild local infusion reactions. Other immediate adverse events were tachycardia (n = 4) and abdominal cramps (n = 1). Four patients developed local thrombophlebitis within the first week and two had transient mild eye floaters during infusion, with no subsequent visual abnormalities. One patient developed central retinal vein occlusion at T3, with complete resolution at T6.

### Insulin dose, glycemic control and GADA status in ASC + VIT D group

In those who received ASC + VIT D, the mean insulin dose at baseline was 0.31 ± 0.26 UI/kg. Insulin dose/kg remained stable at T6 compared to T0 (p = 0.44), except for one patient who became insulin free for 4 months. Insulin doses/kg at T1, T3 and T6 were 0.26 ± 0.21 UI/kg, 0.25 ± 0.17 UI/kg and 0.28 ± 0.14 UI/kg, respectively. After intervention, there was a decrease in HbA1c (7.77 ± 1.14% at T0, 6.21 ± 0.49% at T3 and 6.56 ± 0.66% at T6; T0 vs. T6 p = 0.018). Mean GADA titers remained stable throughout the study (227.65 ± 107.94 units/mL at T0 and 228.51 ± 125.96 units/mL at T6; p = 0.91).

### Evaluation of pancreatic function in ASC + VITD group

**Figure 1:**
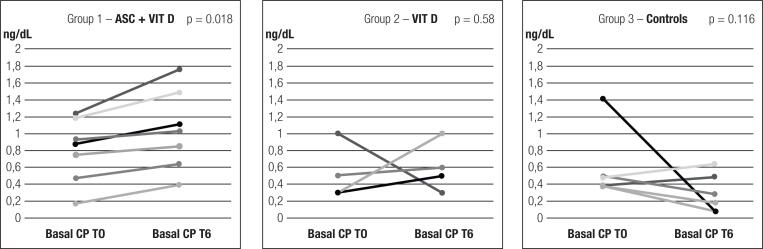
Basal C peptide distribution at T0 and T6 in each group.

All patients who received ASC + VITD had an increase in basal CP 6 months after intervention (T0 = 0.80 ± 0.38 ng/dL; T1 = 0.86 ± 0.48 ng/dL; T3 = 0.74 ± 0.28 ng/dL; T6: 1.04 ± 0.47 ng/dL; T0 vs. T6 p = 0.018), as shown in Figure 1. The mean increase was 40.41 ± 40.79%.

Individual patient basal C-peptide before and 6 months after intervention for each group (group 1: ASC + VIT D; group 2: VIT D; group 3: Controls). CP: c peptide; T0: basal; T6: after 6 months; VIT D: vitamin D; ASC: adipose tissue-derived stem/stromal cells.

**Figure 2:**
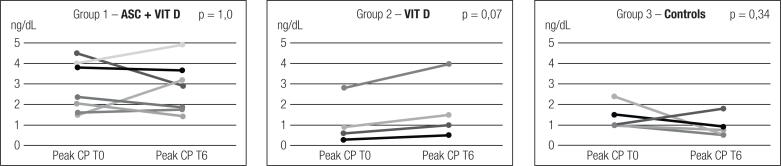
Peak C peptide distribution at T0 and T6 in each group.

Both peak CP and AUC after MMT remained stable 6 months after ASC + VIT D (p = 1.0 and p = 0.62). Peak CP before intervention, at T3 and at T6 were 2.83 ± 1.24 ng/dL, 2.65+/-1.46 ng/dL and 2.82 ± 1.24 ng/dL, respectively (Figure 2). AUC before intervention and at T1, T3 and T6 were 237.08 ± 95.18 ng/mL, 256.82 ± 138.4 ng/mL, 225.92 ± 98.30 ng/mL and 250.26 ± 92.38 ng/mL, respectively. Four patients had increases in peak CP (57.1%), and 5 (71.4%) had an increase in AUC at T6.

Individual patient peak C peptide before and 6 months after intervention for each group (group 1: ASC + VIT D; group 2:VIT D; group 3: Controls). CP: c peptide; T0: basal; T6: after 6 months; VIT D: vitamin D; ASC: adipose tissue-derived stem/stromal cells.

There was an inverse correlation between HbA1c and either peak CP (r = −0.821, p = 0.023) or CP AUC (r = −0.929, p = 0.003) at T6, but not with basal CP (p = 0.25). There was no correlation between insulin dose/kg and basal CP, peak CP or CP AUC at T6 (p = 0.7, p = 0.33, and p = 0.64, respectively).

### Comparison of ASC + VIT D (group 1) with a previous study with VIT D (group 2) and controls (group 3)

Data from patients who received ASCs + VIT D (group 1) were compared with data from a previous study (16) where a group received 2,000 UI VIT D (group 2) and another remained on standard insulin treatment (group 3). Clinical data from all 3 groups are described in Table 2.

**Table 2:** Age, insulin dose, Hba1c and basal and peak C peptide in group 1 (ASC + VIT D), group 2 (VIT D) and group 3 (controls)

	ASC + VIT D Group 1 (n = 7)	VIT D Group 2 (n = 4)	Controls Group 3 (n = 6)	p value *
Age (years)	27.14 ± 6.49	20.75 ± 6.18	19.17 ± 4.02	p = 0.09
Female/male	4 (57.1%)/3 (42.9%)	2 (50%)/2 (50%)	3 (50%)/3 (50%)	p = 0.79
Insulin dose/kg T0	0.31 ± 0.26 UI/kg	0.46 ± 0.25 UI/kg	0.57 ± 0.31 UI/kg	p = 0.9
Insulin dose/kg T6	0.28 ± 1.44 UI/kg	0.55 ± 0.31 UI/kg	0.63 ± 0.28 UI/kg	p = 0.89
Absolute insulin alteration	-0,028 ± 0,21 UI/kg	0.08 ± 0.15 UI/kg	0.06 ± 0.25 UI/kg	p = 0.9
% of insulin dose alteration	15.39 ± 51.67%	20.26 ± 30.45%	23.53 ± 63.80%	p = 0.89
HbA1c T0	7.77 ± 1.14%	9.15 ± 3.05%	8.08 ± 2.4%	p = 0.89
HbA1c T3	6.2 ± 0.49%	6.65 ± 1.03%	8,33 ± 3.25%	p = 0.38
HbA1c T6	6.55 ± 0.66%	6.37 ± 0.62%	7.87 ± 2.01%	p = 0.379
Basal CP T0 (ng/dL)	0.80 ± 0.38	0.52 ± 0.33	0.59 ± 0.39	p = 0.473
Basal CP T6 (ng/dL)	1.04 ± 0.47	0.60 ± 0.29	0.31 ± 0.22	**p = 0.028**
Absolute basal CP modification (ng/dL)	0.23 ± 0.14	0.07 ± 0.57	- 0.28 ± 0.52	**p = 0.045**
% basal CP modification	40.41 ± 40.79	62.41 ± 127.08	33.15 ± 51.6	p = 0.107
Peak CP T0	2.83 ± 1,24	1.27 ± 1.02	1.29 ± 0.58	**p = 0.031**
Peak CP T6	2.82 ± 1,24	1.95 ± 1.38	0.90 ± 0.48	**p = 0.011**
Absolute peak CP modification (ng/dL)	-0.007 ± 1.08	0.67 ± 0.36	-0.39 ± 0.89	p = 0.16
% peak CP modification	8.69 ± 53.1	59.66 ± 11.38	-17.72 ± 55.65	p = 0.079

ASC: adipose tissue-derived stem/stromal cells; VIT D: cholecalciferol; Hba1c: glycated hemoglobin; CP: C peptide; T0: before intervention; T6: 6 months after intervention; group 1: ASC + VIT D; group 2: VIT D; group 3: Controls. Basal CP, Peak CP and insulin dose in two by two comparisons are described in supplementary Figures 2 and 3 (Figures S2 and S3).

**Supplementary figure 1 (figure S1).**
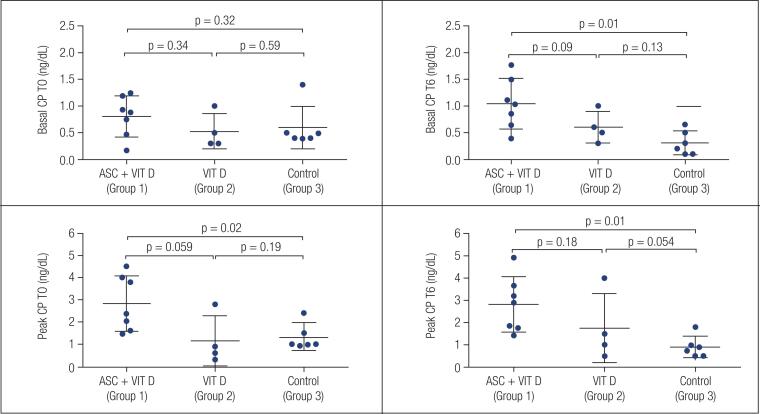
Basal and peak CP before (T0) and 6 months (T6) after ASC infusion and VIT D supplementation.

There was a similar age and gender distribution among groups (Table 2). Basal and peak CP at T0 and T6 in each group are described in Figures 1 and 2. Group 1 had an increase in basal CP over 6 months (p = 0.018), but basal CP remained stable in groups 2 and 3 (p = 0.58 and p = 0.116, respectively), as shown in Figure 1. In Table 2, there was a difference in basal CP at T6 (p = 0.028) comparing all three groups. For two-by-two comparisons, the difference was seen only between groups 1 and 3, as shown in supplementary Figure 1 (Figure S1). An increase in basal CP was observed in all patients in group 1 vs. 3/4 (75%) and 2/6 (33.3%) patients in groups 2 and 3, respectively (p = 0.011). The percentage of increase in basal CP was higher in patients who received any intervention (ASCs + VIT D or VIT D) than in others (+48.41 ± 77.24 vs. −33.15 ± 51.60%; p = 0.035). No differences were found in percentage of basal CP increase from T0 to T6 between the three groups, as shown in Table 2 (p = 0.107). Two-by-two comparisons did not show differences between groups in the percentage of CP alteration from T0 to T6 after Bonferroni correction (p = 0.035, p = 0.257 and p = 1.0 for ASC + VIT D vs controls, VIT D vs controls and ASC + VIT D vs. VIT D, respectively).

Peak CP remained stable after 6 months in all 3 groups as described in Figure 2. In Table 2, there was a difference in peak CP at T0 and T6, comparing the three groups (p = 0.031 and p = 0.011, respectively). In two-by-two comparisons, the difference was significant only between groups 1 and 3 at T6 (p = 0.01) (Supplementary Figure 1; Figure S1). The percentage of increase in peak CP did not differ between groups, p = 0.079 (Table 2). There was no difference between groups in the frequency of individuals who had an increase in peak CP after 6 months (p = 0.44).

**Figure 3:**
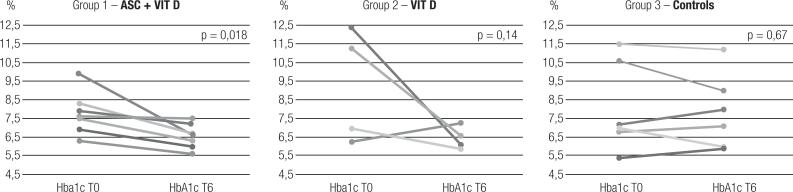
Hba1c at T0 and T6 in each group.

HbA1c improved in group 1 after 6 months (7.77 ± 1.14% vs. 6.55 ± 0.66, p = 0.018), but had no difference in group 2 (p = 0.14) and group 3 (p = 0.67), as shown in Figure 3. Insulin dose/kg was similar before and after intervention in all groups (group 1 T0 = 0.31 ± 0.26 UI/kg vs. T6 = 0.28 ± 1.44 UI/kg, p = 0.44; group 2 T0 = 0.46 ± 0.25 UI/kg vs. T6 = 0.55 ± 0.31 UI/kg, p = 0.28; group 3 T0 = 0.57 ± 0.31 UI/kg vs. T 6= 0.63 ± 0.28 UI/kg, p = 0.46). Two-by-two comparisons between groups are described in supplementary Figure 2 (Figure S2). Percentage of insulin dose increase after 6 months was similar between groups (p = 0.89).

Individual patient basal C-peptide before and 6 months after intervention for each group (group 1: ASC + VIT D; group 2: VIT D; group 3: Controls). CP: c peptide; T0: basal; T6: after 6 months; VIT D: vitamin D; ASC: adipose tissue-derived stem/stromal cells.

At T6, all patients (100%/n = 7) in group 1 were in a honeymoon period (insulin dose ≤ 0.5 UI/kg with HbA1c < 7.5%) vs. 75% (n = 3/4) in group 2 and 50% in group 3 (n = 3/6), p = 0.01.

At T0, vitamin D levels did not differ between groups (33.06 ± 13.55 ng/mL,27.85 ± 11.17 ng/mL, 24.1 ± 8.51 ng/mL for groups 1, 2 and 3, respectively, p = 0.26). At T6, groups 1 and 2 had similar vitamin D levels (45.0 ± 14.63 vs. 55.89 ± 15.78; p = 0.41), which were higher than in group 3 (28.33 ± 5.44; p = 0.002). There was no correlation between basal or peak CP and vitamin D levels in those patients who received oral VIT D (p = 0.39 and p = 0.86, respectively).

## DISCUSSION

This study evaluated the safety and efficacy of allogenic ASCs without immunosuppression associated with 2,000 UI VIT D supplementation for 6 months in patients with recent-onset T1D. After intervention, all patients had a good glycemic control and low insulin requirements, without significant decline in β-cell function, with few or transient complications.

This was the first trial to use allogenic ASCs without immunosuppression in patients with T1D of short duration. Other authors have investigated the role of adult stem cells from different origins in the preservation of β-cells for patients with T1D. HSC transplantation with cyclophosphamide and anti-thymoglobulin led to significant increase in CP and transient insulin independence, but the potential toxicity of immunosuppressive agents limits their widespread clinical use (9). Carlsson and cols. evaluated the efficacy of autologous bone marrow MSC in recent onset T1D patients (21). Both AUC and peak CP improved after 1 year when compared to controls with no adverse events. As bone marrow is not easily accessed, subcutaneous adipose tissue might be a more interesting source of MSC (13). Although Thakkar and cols. have tested allogenic and autologous ASC for patients with T1D, the study population comprised individuals with long-standing disease, concomitant use of immunosuppressive agents, bone marrow transplantation and ASC culture to generate insulin-secreting cells in vivo and intraportal infusion (22).

Allogenic ASC was used in this trial. Differently from autologous cells, they could potentially replace the autoreactive host immune system with a more tolerant donor profile (23). There is concern whether the immune properties of the MSC are preserved in individuals with diabetes, which favors the use of allogenic cells for transplantation in this scenario. The safety and promising outcome of this clinical trial using allogenic cells encourages the generation of master banks (cryopreserved cells) for cell therapy. ASC can be isolated from young, healthy donors, fully characterized, expanded in vitro and cryopreserved for their use in a range of diseases, including T1D.

Therapy with ASC was safe and led to few or transient adverse events. Most patients presented tachycardia during infusion, which resolved soon after its suspension. Transient thrombophlebitis was also frequent. These events might have been associated with high cellularity concentration, high viscosity, or other cell stabilizing products. One patient presented central vein occlusion 3 months after infusion, with complete resolution. This was probably not associated with the therapy since it occurred months after the intervention.

Six months after intervention, there was an absolute increase in basal CP in the group that received ASC + VIT D. As basal endogenous insulin secretion has a micropulsatile pattern and may have paracrine effects in the regulation of glucagon secretion, it is possible that this improvement might influence glycemic control during a fasting state (24,25). Peak CP and CP AUC remained stable in this period after the intervention with HbA1c within target and low insulin requirements. Although these findings could represent a honeymoon state, previous data from the Trialnet and others indicate that there is a decrease in CP AUC and peak CP in patients with similar ages in the first 6-12 months of T1D (26,27). However, most patients included in Trialnet were Caucasian, and there is scarce information about the progression of the β-cell function in T1D from multiethnic populations.

In order to determine whether the favorable evolution of β-cell function was related to the treatment, we compared data from patients who underwent ASC infusion + VIT D with individuals who received standard insulin treatment in this study or in a previous analysis of a similar population, as well as with individuals that received only VIT D as intervention (16). The improvement in basal CP observed in patients that received ASC + VIT D was not seen in the other groups. A difference in basal CP at T6 was observed among the 3 groups, but in two-by-two comparisons the difference was seen only between group 1 (ASC infusion + VIT D) and 3 (controls). Although this suggests that treatment with ASC + VIT D is associated with better basal CP evolution, this information should still be interpreted cautiously, due to the limited number of patients in each group, the absence of differences in the absolute or percentage of basal CP difference between groups as well as the lack of peak CP alterations or differences in the whole sample, which is a more traditional and reliable marker for β-cell function.

Patients in group 1 had an improvement in HbA1c without a significant increase in insulin dose/kg, which might be secondary to the increase in basal insulin secretion observed in those patients. Moreover, all patients in ASC + VIT D therapy group were in honeymoon phase after 6 months, which was superior to that observed in groups that used vitamin D without ASC (3/4) and conventional therapy (3/6).

This study indicates a potential benefit of the combination of peripheral ASC infusion + oral VIT D supplementation for patients with the recent onset of T1D. The infusion of these cells in the peripancreatic area might have more pronounced effects, as it has been previously shown that part of the stem cells that are infused in peripheral veins migrates to the lungs, which could compromise their immunomodulatory action (28,29). Moreover, it is possible that the slightly beneficial effects of stem cells for patients with the recent onset of T1D observed in our group, when compared to more striking results observed in former studies with hematopoietic stem cells (9), might have been due to concomitant immunosuppression used in those. In this study, we administered ASC without immunosuppressive agents to avoid their toxicity (9).

This study has some limitations. First, the limited number of patients might have influenced the results. However, this was a pilot study with the priority of establishing the safety of ASC. As therapy has proven to be safe, further, larger studies may be developed. Second, it is not possible to determine whether the beneficial effect of ASC in pancreatic function was due to immune modulation or secondary to their differentiation in beta cells. Moreover, this was an open study, and most participants accepted entrance only in the intervention arm. Therefore, comparison between individuals that received ASC + VIT D with patients in standard insulin treatment depended mostly on a previous study performed on a similar population. Finally, a longer follow-up is necessary to investigate the long-term safety and efficacy of ASC + VIT D. However, this was the first study to show the safety of allogenic ASC without immunosuppression in patients with T1D of short duration, which opens a new possibility for clinical trials.

To conclude, therapy with allogenic ASC + VIT D without immunosuppression was safe and might have a role in the preservation of β-cells in patients with recent-onset T1D.

## Figures and Tables

*P value with Bonferroni correction, level of significance of 0.017.

**Supplementary figure 2 (figure S2):**
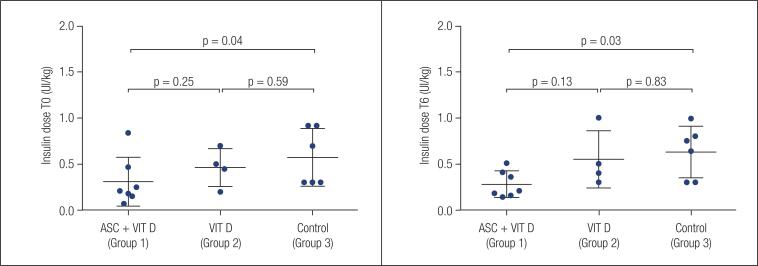
2. Insulin dose/kg before (T0) and 6 months after ASC infusion and VIT D supplementation.

Figure 2 describes insulin dose/kg before (T0) and 6 months (T6) after intervention for each group (group 1: ASC + VIT D; group 2: VIT D; group 3: Controls); T0: basal; T6: after 6 months; VIT D: vitamin D; ASC: adipose tissue-derived stem/stromal cells. P values represent comparisons two-by-two.

*P value with Bonferroni correction, level of significance of 0.017.

## Appendix

**Supplementary table 1:** Flow cytometer

Marker	CD 105	CD 73	CD 90	CD 29	CD 166	CD 44	CD 36	CD 14	CD 34	CD 45	CD 19	HLA-DR	CD 31	CD 106
Means	50,42%	51,16%	49,66%	47,45%	46,09%	12,80%	5,92%	0,71%	0,67%	0,71%	0,50%	0,57%	2,39%	3,77%
DP	0,055	0,415	0,434	0,442	0,467	0,395	0,131	0,038	0,005	0,007	0,003	0,005	0,002	0,039

Data are means ± SD (95% CI).

FITC-labeled CD14 (BD#555397), CD45 (BD#555482), CD19 (BD#555412), CD44 (BD#555478); PE-labeled CD73 (BD#550257), CD90 (BD#555596), CD166 (BD#559263), PerCP-labeled HLA-DR (BD#551375); APC-labeled CD34 (BD#555824), CD105 (BD#562408), CD29 (BD#559883) all purchased from BD (Pharmingen). At least 100.000 events were acquired on a BD FACSCalibur™ flow cytometer (BD Biosciences), and data were analyzed using FlowJo 10 (TreeStar) software11A.
